# Implications of dynamic changes in miR-192 expression in ischemic acute kidney injury

**DOI:** 10.1007/s11255-016-1485-7

**Published:** 2016-12-29

**Authors:** Lulu Zhang, Yuan Xu, Song Xue, Xudong Wang, Huili Dai, Jiaqi Qian, Zhaohui Ni, Yucheng Yan

**Affiliations:** 1 Department of Nephrology, Molecular Cell Lab for Kidney Disease, Renji Hospital, School of Medicine, Shanghai Jiao Tong University, 160 Pujian Road, Shanghai, 200127 China; 2 Department of Cardiac Surgery, Renji Hospital, School of Medicine, Shanghai Jiao Tong University, 160 Pujian Road, Shanghai, 200127 China

**Keywords:** miR-192, Acute kidney injury, Cardiac surgery, microRNAs, Ischemia–reperfusion injury

## Abstract

**Purpose:**

Ischemia–reperfusion injury (IRI) is a major cause of acute kidney injury (AKI) with poor outcomes. While many important functions of microRNAs (miRNAs) have been identified in various diseases, few studies reported miRNAs in acute kidney IRI, especially the dynamic changes in their expression and their implications during disease progression.

**Methods:**

The expression of miR-192, a specific kidney-enriched miRNA, was assessed in both the plasma and kidney of IRI rats at different time points after kidney injury and compared to renal function and kidney histological changes. The results were validated in the plasma of the selected patients with AKI after cardiac surgery compared with those matched patients without AKI. The performance characteristics of miR-192 were summarized using area under the receiver operator characteristic (ROC) curves (AUC-ROC).

**Results:**

MiRNA profiling in plasma led to the identification of 42 differentially expressed miRNAs in the IRI group compared to the sham group. MiR-192 was kidney-enriched and chosen for further validation. Real-time PCR showed that miR-192 levels increased by fourfold in the plasma and decreased by about 40% in the kidney of IRI rats. Plasma miR-192 expression started increasing at 3 h and peaked at 12 h, while kidney miR-192 expression started decreasing at 6 h and remained at a low level for 7 days after reperfusion. Plasma miR-192 level in patients with AKI increased at the time of ICU admission, was stable for 2 h and decreased after 24 h. AUC-ROC was 0.673 (95% CI: 0.540–0.806, *p* = 0.014).

**Conclusions:**

Plasma miR-192 expression was induced in a time-dependent manner after IRI in rats and patients with AKI after cardiac surgery, comparably to the kidney injury development and recovery process, and may be useful for the detection of AKI.

## Introduction

Acute kidney injury (AKI) is one of the most common complications in patients with prolonged hospital stays, increasing medical costs and poor outcomes [1]. Ischemia–reperfusion injury (IRI) is a major cause of AKI [1, 2], characterized by acute tubular necrosis [3]. However, the mechanisms underlying AKI development are unclear and diagnostic biomarkers have not been established, leading to the lack of specific treatment [4]. Recent studies identified proteins, including KIM-1 [5], NGAL [6] and IL-18 [7], as potential biomarkers in ischemic AKI. However, none of them has been validated or is routinely used in the clinic. Thus, the discovery and validation of additional reliable biomarkers are strongly needed to detect AKI [8].

MicroRNAs (miRNAs) are short, endogenous non-coding RNAs, 18–22 nt long, that regulate gene expression at the post-transcriptional level by targeting messenger RNAs and inhibiting translation [9]. MiRNAs have important functions in numerous biological processes [9]. Owing to their stability in the blood, urine and other body fluids, miRNAs are considered novel biomarkers in various diseases [10–12], including acute and chronic kidney diseases [13, 14]. Johan et al. determined that circulating miR-210 levels significantly increased in critically ill patients with AKI and was an independent and powerful predictor of 28-day survival [15]. A study on 120 adult patients undergoing cardiac surgery showed that miR-21 levels in the urine and plasma were upregulated in AKI patients and both were associated with AKI progression [16]. The areas under the curve (AUCs) for urine and plasma miR-21 associated with established AKI were 0.68 (95% CI: 0.59–0.78) and 0.80 (95% CI: 0.73–0.88), respectively [16]. However, no study evaluated variations of these miRNAs over time. Levels of specific miRNAs might vary during disease progression, and early detection of AKI is important for intervention. We postulated that dynamic changes in the levels of plasma miRNAs might be involved in the development of IRI and might be useful for the detection of AKI.

In the present study, we analyzed miR-192 expression levels in the plasma and kidney tissues from IRI rats after miRNA profiling and verified its changes in the plasma of patients after cardiac surgery at different time points to determine whether miR-192 can be used as a tool to detect IRI kidney injury.

## Materials and methods

### Ethics statement

All animal experiments were approved by the Animal Care and Use Committee of Ren Ji Hospital and carried out in accordance with the National Institutes of Health Guide for Use of Laboratory Animals. The human study was approved by the Ethical Committee of Ren Ji Hospital and in accordance with the principle of the Declaration of Helsinki. In addition, all patients signed informed written consent before cardiac surgery.

### Animal model

Male Sprague–Dawley (SD) rats, weighing 150–200 g, were purchased from the SLAC Laboratory Animal Co. Ltd (Shanghai, China) and housed in animal cages with suitable conditions in the Animal Department of Renji Hospital. Rats were randomly grouped into the sham or IRI group. In miRNA PCR array and miR-192 validation study, nine rats in each group were euthanized after 24 h of reperfusion. In the time-course study, six rats from each group were euthanized 1, 3, 6, 12, 24 h, 3 or 7 days after surgery. The IRI procedures were performed as previously described [17]. Blood was collected from the abdominal aorta and placed into EDTA anti-coagulation tubes. Once harvested, one quarter of each kidney was placed into 10% formalin for histological examination, while the remaining was stored at −80 °C after being snap-frozen in liquid nitrogen for other studies.

### Patients

Ninety-three patients aged 40–80 years, undergoing open cardiac surgeries in the Cardiac Surgery Division of Ren Ji Hospital from January 2014 to October 2014, were enrolled. All the patients with pre-existing end-stage renal disease requiring renal replacement therapy (RRT), cancer, diabetes, lupus nephritis, infection diseases, previous cardiac interventional therapy or minimally invasive cardiac surgery were excluded. Among them, 35 (37.6%) patients developed AKI, including 26 (28.0%) of Stage 1, 5 (5.4%) of Stage 2 and 4 (4.3%) of Stage 3. Thirty-five patients who developed AKI during the follow-up and thirty-five controls who also underwent open cardiac surgery, but did not develop AKI were matched at a 1:1 ratio based on age, sex, comorbidities and type of surgery in the subsequent study. The AKI diagnosis was defined by the Kidney Disease Improving Global Outcomes (KDIGO) criteria with a postoperative increase in plasma creatinine ≥50% from baseline or ≥0.3 mg/dL [18]. Patients who presented hemolysis in the plasma samples at two or more time points were also excluded. Plasma samples were collected at different time points, including preoperation, 0, 2, 24 and 72 h after admission to the intensive care unit (ICU).

### Total RNA extraction

Plasma was obtained within 1 h by two-step centrifugation at 3000*g* at 4 °C for 15 min, followed by that at 12,000*g* at 4 °C for 15 min. The kidney homogenates were obtained by adding 400 μL of lysis buffer. RNA extraction was performed with TaqMan™ mirVana™ PARIS kit (ThermoFisher, Waltham, MA, USA) according to the manufacturer’s instructions.

### MiRNA PCR array assay

MiRNAs were assessed by rodent TaqMan low-density array (TLDA) assay (ThermoFisher) according to the manufacturer’s instructions. An equal amount of three randomly selected samples was used to generate the pooled sample. Six pooled samples were obtained, three from each group. RNA was extracted from the six pooled samples. Reverse transcription, preamplification and PCRs were performed following the manufacturers’ protocols.

### Quantitative real-time PCR

Real-time PCR was performed to validate rat and human plasma miRNA using TaqMan^®^ microRNA reverse transcription kit (ThermoFisher), TaqMan^®^ Universal Master Mix II, no UNG (ThermoFisher) and TaqMan^®^ microRNA assay (ThermoFisher) according to the manufacturer’s instructions. The 2^(−ΔΔ*Ct*)^ value was calculated. Cel-mir-39 was used as a spike-in control for plasma miRNA normalization, while U6 was used as the internal control for rat tissue miRNA normalization.

### Biochemical tests

Plasma urea nitrogen (BUN) and creatinine were detected by enzymatic method, using an automatic biochemical analyzer.

### Histological examination

Histological examination was performed as previously described [19]. Tubular damage was scored by calculating the percentage of tubules in the corticomedullary junction that displayed tubular epithelia cell degeneration, cell necrosis, loss of the brush border, formation of lumen casts and tubular dilatation, as follows: 0, none; 1, ≤10%; 2, 11–25%; 3, 26–45%; 4, 46–75%; and 5, >75%. One blinded observer assessed the tubular damage. Ten high-power fields (magnification, 200×) were examined for each rat.

### Statistical analysis

All statistical analyses were performed with SPSS 16.0 software (SPS, Chicago, IL, USA). Continuous data were presented as mean ± standard deviation and compared by using Student’s *t* test. Non-continuous data were presented as median (interquartile) and compared using Mann–Whitney U test. To compare categorical variables, we performed the *χ* ^2^ or Fisher’s exact test, as appropriate. Multiple comparisons of variables within and between two groups of patients were compared by two-way ANOVA followed by post hoc nonparametric statistics. The AUC was calculated from a standard receiver operating characteristic (ROC) curve of microRNA for predicting AKI. *p* < 0.05 was considered statistically significant.

## Results

### Bilateral kidney ischemia–reperfusion induced an increase in plasma BUN, creatinine and tubular damage in a rat model

**Fig. 1:**
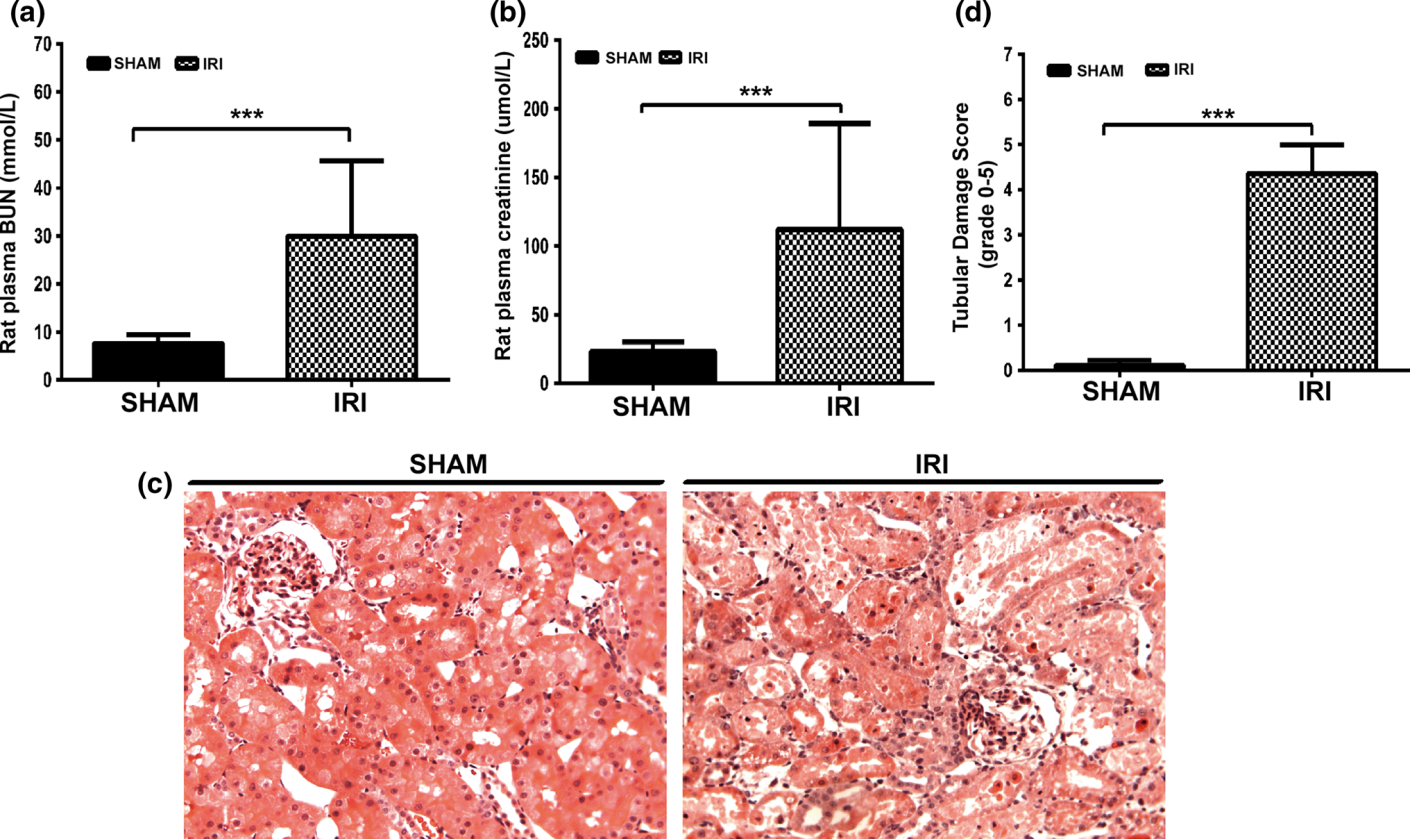
Ischemia–reperfusion-induced kidney functional and structural injury. Plasma BUN (**a**) and creatinine (**b**) levels, histological examination (**c**, magnification, ×200), tubular damage score (**d**) in IRI and sham rats. ***p < 0.001 versus the sham group

To determine the miRNA expression profile related to tissue injury and repair processes following AKI, we used a well-established model of bilateral renal IRI. After 45 min of ischemia and 24-h reperfusion, plasma BUN and creatinine were significantly elevated in the IRI group. In the sham group, BUN and creatinine were 7.67 ± 1.73 mmol/L and 23.3 ± 7.02 μmol/L, respectively. In the IRI group, BUN and creatinine increased to 29.95 ± 15.31 mmol/L and 112.05 ± 75.22 μmol/L, respectively (Fig. 1a, b). The histological staining further supported kidney injury. Significant tubular injury was observed in IRI rats presenting extensive tubular necrosis, loss of the brush border, cast formation and tubular dilatation in the corticomedullary junction (Fig. 1c, d).

### Global microRNA profiling of IRI rats

**Fig. 2:**
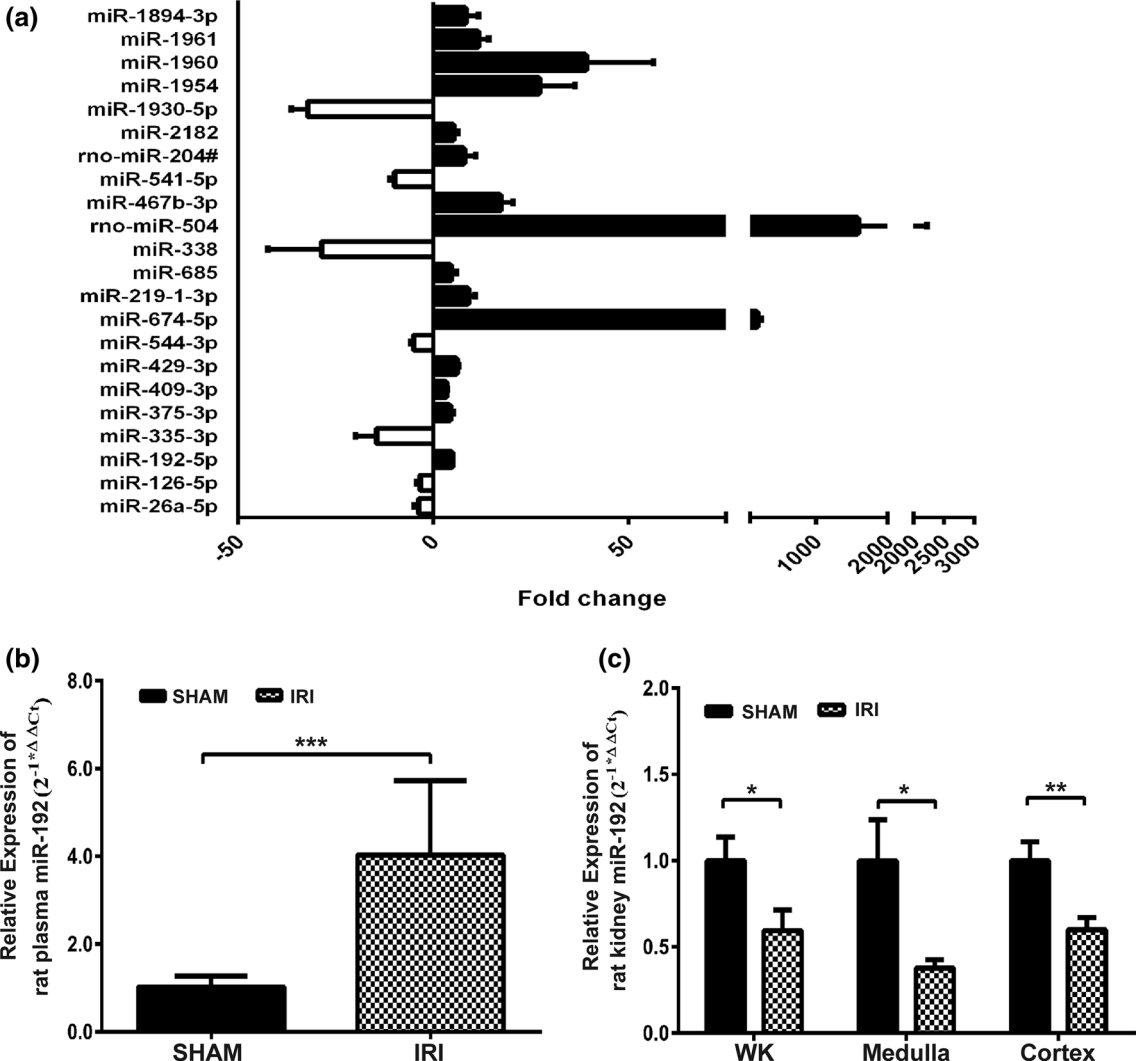
Global profiling and validation of plasma miRNAs in IRI rats. Rat plasma miRNA profiling **a** showing 22 differentially expressed miRNAs (fold change > 3; *p* < 0.05) in IRI rats. **b** Plasma and **c** renal tissue miR-192 levels in the IRI and sham groups. WK, whole kidney, *p < 0.05, **p < 0.01, ***p < 0.001 versus the sham group

Forty-two miRNAs differentially expressed were identified by rat plasma global miRNA PCR array, among which 22 were aberrantly expressed with more than threefold change between groups (Fig. 2a). There were fifteen elevated miRNAs and seven downregulated miRNAs.

### Validation of altered microRNAs in the plasma and kidney tissues of IRI rats

Among the identified miRNAs, miR-192 was preferentially expressed in the kidney and showed more than a threefold change. Therefore, we chose miR-192 for further validation. As shown in Fig. 2b, the level of miR-192 in the plasma of IRI rats increased by about fourfold compared to that of the sham group. We further confirmed the expression of miR-192 in kidney tissues. In IRI rats, miR-192 expression was decreased by about 40% in the whole kidney, about 60% in the medulla and about 40% in the cortex, compared to the sham group (Fig. 2c).

### Dynamic changes of plasma and kidney miR-192 expression in IRI rats

**Fig. 3:**
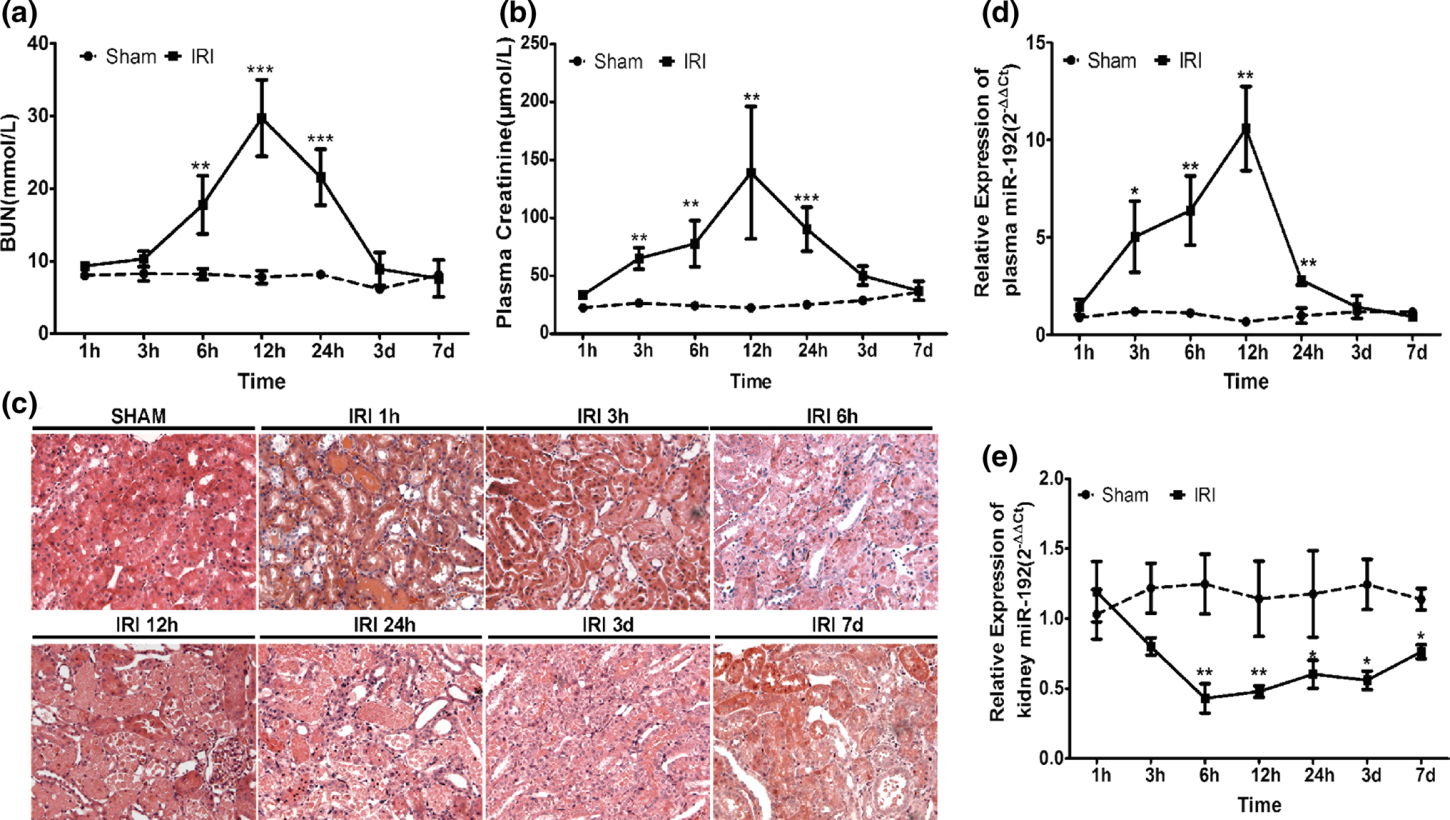
Time course of the IRI-induced dynamic kidney injury and changes of miR-192 in the plasma and kidney tissues of IRI rats. BUN (**a**) and plasma creatinine (**b**) levels in sham and IRI rats. Histological staining (**c**) showed gradually worsening kidney injury with prolonged reperfusion time. Magnification, ×200. Plasma (**d**) and renal tissue (**e**) miR-192 levels in the IRI and sham groups at different time points. *p < 0.05; **p < 0.01; ***p < 0.001 versus the sham group

To investigate how early miR-192 was changed after renal IRI, we observed the dynamic expression of miR-192 in the plasma and kidney tissues of IRI rats. BUN was significantly increased at 6 h after reperfusion, peaked at 12 h and returned to normal levels at 7 day of IRI (Fig. 3a). Plasma creatinine was significantly increased at 3 h after reperfusion, continued to increase at 12 h, finally returned to baseline at 3 day after IRI (Fig. 3b). H&E staining confirmed kidney injury (Fig. 3c). Levels of plasma and kidney miR-192 were assayed at 1, 3, 6, 12, 24 h, 3 and 7 day after 45 min of ischemia. Plasma miR-192 expression was elevated at 3 h after reperfusion, peaked at 12 h and returned to baseline at 3 day after reperfusion (Fig. 3d). However, the time-course study showed significantly decreased levels of miR-192 in kidney tissues of IRI rats, with an initial decrease at 6 h and a continuous low level until 7 day after reperfusion (Fig. 3e).

### Circulating levels of miR-192 increased in patients after cardiac surgery

**Fig. 4:**
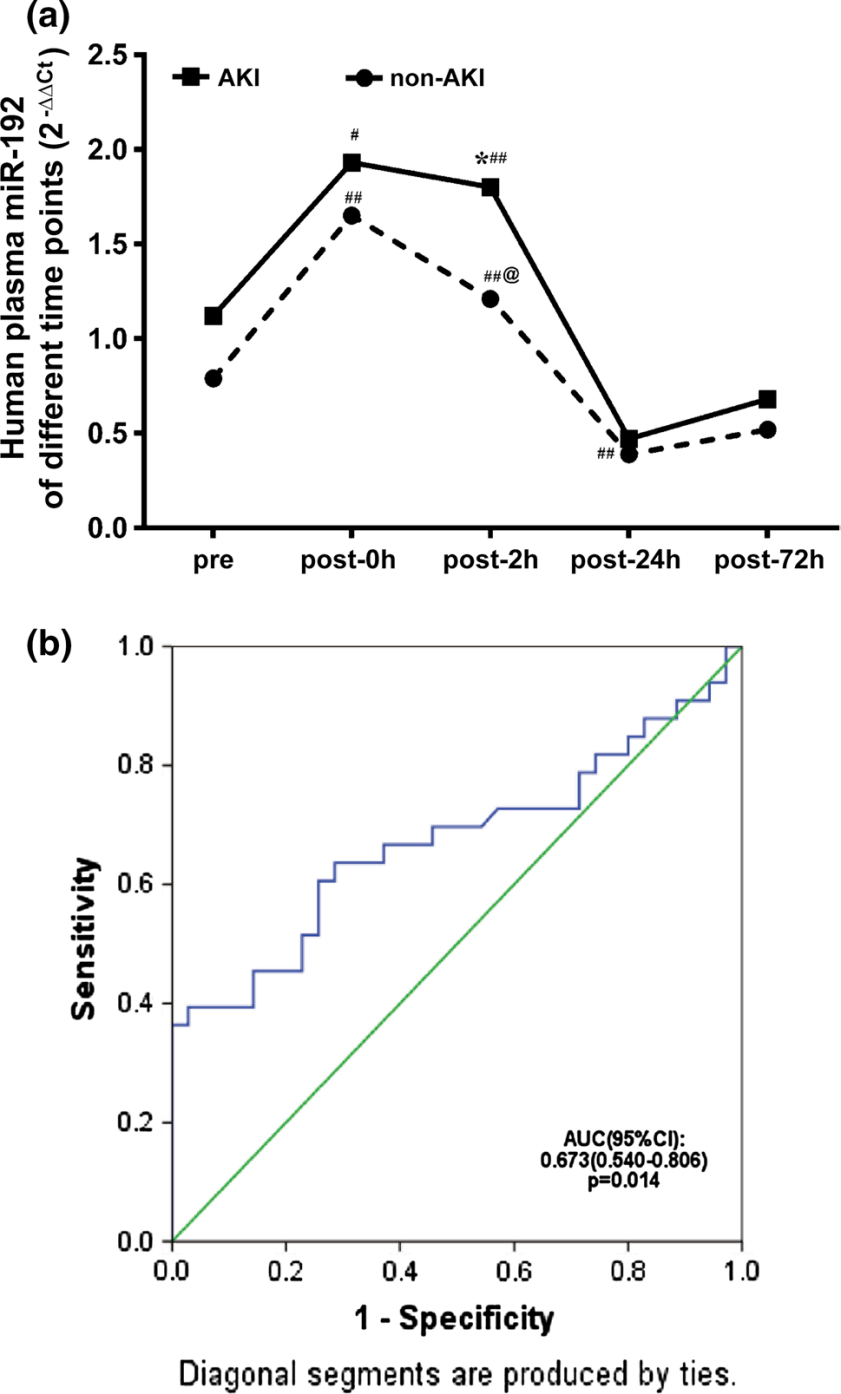
Plasma miR-192 expression of patients in the AKI group varied over time and could predict AKI incidence after cardiac surgery. Data are expressed as the median (interquartile). **a** The miR-192 expression of patients ^#^ *p* < 0.05, ^##^ *p* < 0.01 versus prelevels; *p < 0.05 versus the non-AKI group; ^@^ *p* < 0.05 versus post 0 h. **b** The area under the ROC curve of post 2 h miR-192

To investigate the relationship between miR-192 and AKI, we observed the dynamic changes of plasma miR-192 levels in 70 patients who underwent cardiac surgery, 35 patients with AKI and 35 patients without AKI. As shown in Fig. 4a and Table 1, plasma miR-192 levels increased rapidly at 0 h after admission to the ICU and were stable between 0 and 2 h after surgery in patients who developed AKI. MiR-192 level at 24 and 72 h was similar to that of preoperation. The expression pattern of miR-192 in the non-AKI group was similar to that of the patients with AKI, except that miR-192 levels decreased at 2 h, but was still higher than that before operation. MiR-192 levels at 2 h after ICU admission in the AKI group were significantly higher than those in the non-AKI group. ROC curve analysis showed that miR-192 could be used to diagnose AKI (Fig. 4b). The area under the ROC curve (AUC-ROC) was 0.673 (95% CI: 0.540–0.806, *p* = 0.014) for plasma miR-192 at 2 h after ICU admission, indicating that plasma miR-192 can be an early marker for the detection of established AKI. When the cutoff value was 1.445, the sensitivity and specificity were 66.7 and 62.9%, respectively.

**Table 1:** Plasma miR-192 levels of patients at different time points

	Non-AKI	AKI	*p* value (AKI vs. non-AKI)
Pre	0.79 (0.41, 1.16)	1.12 (0.47, 2.42)	0.092
0 h	1.65 (0.97, 2.36)^###^	1.93 (0.93, 3.24)^##^	0.277
2 h	1.21 (0.88, 1.77)^##^	1.80 (0.94, 5.10)^##^	0.014
24 h	0.39 (0.28, 0.67)^##^	0.47 (0.29, 1.83)*^#^	0.196
72 h	0.52 (0.22, 0.98)	0.68 (0.23, 1.73)	0.165

*AKI* acute kidney injury

* *p* < 0.05 versus non-AKI patients

^*#*^ *p* < 0.05, ^##^ *p* < 0.01, ^###^ *p* < 0.001 versus prelevels

### Baseline characteristics of patients after cardiac surgery

Patient characteristics are shown in Table 2. Of the 70 patients studied, 43 were males and 35 developed AKI after surgery. The mean patients’ age was 64.31 ± 8.17 years, and the preoperative plasma creatinine was 74.97 ± 16.25 μmol/L. Other characteristics of patients with or without AKI are shown in Table 2. There was no significant difference in sex, age, plasma creatinine level and comorbidities, including chronic kidney disease (CKD), hypertension, anemia and use of contrast medium and angiotensin-converting enzyme inhibitors/angiotensin II receptor blocker (ACEI/ARB). The intraoperative variables, including the proportion of surgery type, operation time and cardiopulmonary bypass (CPB), were also similar. Coronary artery bypass graft (CABG) surgeries included on-pump CABG and off-pump CABG. Five patients underwent off-pump CABG and 3 of them developed AKI. The postoperative variables, including hypotension, low blood volume symptom and congestive heart failure, were comparable between groups. Seven patients developed an infection during the follow-up; one developed an upper respiratory tract infection, while the others developed lung infections. One patient needed RRT after surgery, and the incidence of RRT was not statistically significant. All the three patients who died were in the AKI group.

**Table 2:** Patient characteristics

	All (*n* = 70)	Non-AKI (*n* = 35)	AKI (*n* = 35)	*p* value (AKI vs. non-AKI)
Preoperative variables				
Male *n* (%)	43 (61.4%)	21 (60.0%)	22 (62.9%)	0.806
Age (years)	64.31 ± 8.17	62.66 ± 7.07	65.97 ± 8.93	0.090
Plasma creatinine (μmol/L)	74.97 ± 16.25	72.62 ± 15.36	77.33 ± 16.98	0.259
eGFR (ml/min/1.73 m^2^)	90.64 ± 18.32	93.22 ± 16.71	88.06 ± 19.71	0.242
Body mass index	22.88 ± 3.42	22.81 ± 3.45	22.95 ± 3.43	0.867
Chronic kidney disease *n* (%)	17 (48.5%)	8 (22.9%)	9 (25.7%)	0.780
Hypertension *n* (%)	29 (41.4%)	13 (37.1%)	16 (45.7%)	0.467
NYHA >II *n* (%)	16 (22.9%)	7 (20.0%)	9 (25.7%)	0.428
Use of contrast medium *n* (%)	36 (51.4%)	18 (51.4%)	18 (51.4%)	1.000
Use of ACEI/ARB *n* (%)	27 (38.6%)	14 (40.0%)	13 (37.1%)	0.806
Intraoperative variables				
Type of surgery				
Valve *n* (%)	31 (44.3%)	15 (42.9%)	16 (45.7%)	0.810
CABG *n* (%)	24 (34.2%)	13 (37.2%)	11 (31.4%)	1.000
CABG + valve *n* (%)	9 (12.9%)	4 (11.4%)	5 (14.3%)	1.000
Other operations *n* (%)	6 (8.6%)	3 (8.6%)	3 (8.6%)	1.000
Operation time (h)	5.36 ± 1.39	5.35 ± 1.19	5.38 ± 1.59	0.932
CPB *n* (%)	52 (74.3%)	25 (71.4%)	27 (77.14%)	0.584
CPB time (hours)	1.87 ± 0.85	1.78 ± 0.69	1.95 ± 0.99	0.487
Postoperative variables				
Hypotension *n* (%)	8 (11.4%)	2 (5.7%)	6 (17.1%)	0.223
Low blood volume *n* (%)	9 (12.9%)	2 (5.7%)	7 (20.0%)	0.127
Congestive heart failure *n* (%)	3 (4.3%)	1 (2.9%)	2 (5.7%)	0.958
Infection *n* (%)	7 (10.0%)	3 (8.6%)	4 (11.4%)	0.934
RRT *n* (%)	1 (1.4%)	0 (0%)	1 (2.9%)	0.485
Mortality *n* (%)	3 (4.3%)	0 (0.0%)	3 (8.6%)	0.217

*AKI* acute kidney injury, *eGFR* estimated glomerular filtration rate, *NYHA* New York Heart Association grade for heart failure, *ACEI/ARB* angiotensin-converting enzyme inhibitors/angiotensin II receptor blocker, *CABG* coronary artery bypass graft, *CPB* cardiopulmonary bypass, *RRT* renal replacement therapy

### Dynamic changes of plasma creatinine in patients

As shown in Table 3, the levels of plasma creatinine remained stable after cardiac surgery in the 35 non-AKI patients, except for a small increase at 24 h after admission to the ICU (about 1.15-fold). In the 35 AKI patients, the level of plasma creatinine at 24 and 72 h increased 1.84-fold and 1.49-fold compared to the preoperation levels. Although similar to the preoperation levels, plasma creatinine at 0 and 2 h in AKI patients was a little higher than that in non-AKI patients. The level of creatinine at 24 h after ICU admission in AKI patients was significantly higher than that in non-AKI patients.

**Table 3:** Plasma creatinine levels of patients at different time points

	Non-AKI		AKI	
	Plasma creatinine (μmol/L)	Fold change^a^	Plasma creatinine (μmol/L)	Fold change
Pre	72.62 ± 15.36	/	77.33 ± 16.98	/
0 h	72.46 ± 23.54	1.00	100.09 ± 27.14***	1.31
2 h	68.23 ± 21.26	0.93	96.40 ± 28.20***	1.26
24 h	82.94 ± 18.40^#^	1.15	141.40 ± 55.92***^###^	1.84
72 h	70.88 ± 18.39	0.91	117.54 ± 70.43**^##^	1.49

*AKI* acute kidney injury

* *p* < 0.05, ** *p* < 0.01, *** *p* < 0.001 versus non-AKI patients; ^##^ *p* < 0.01, ^###^ *p* < 0.001 versus prelevels

^a^Fold change, versus prelevels

## Discussion

In the present study, we screened miRNAs in the plasma of IRI rats and identified 42 miRNAs with altered expression, among which 15 miRNAs were upregulated. In the time-course study, miR-192 expression in the plasma and kidney tissues of IRI rats varied in a time-dependent manner. In the clinical study, plasma miR-192 at 2 h after ICU admission presented a modest predictive value for the diagnosis of AKI.

Our study showed dynamic changes of miR-192 expression in the plasma and kidney tissues of IRI rats. These dynamics correlated with histological findings of kidney injury and with increases in concentrations of known markers of renal dysfunction (BUN and creatinine). Thus, miR-192 may reflect kidney IRI. A previous study indicated that plasma miR-192 was upregulated at 6 h after reperfusion in IRI rats [20]. Moreover, the expression pattern of plasma miR-192 was discordant with that of kidney miR-192. Similarly, in a mouse study, Bellinger et al. [21] determined that the expression of few miRNAs changed in a coordinated manner, while that of most miRNAs in an uncoordinated manner. Saikumar et al. [22] demonstrated that kidney miR-21 and miR-155 were upregulated after IRI, while blood miR-21 and miR-155 were downregulated. Mechanistic relationships between renal and circulating miRNAs are unclear, and whether the increased circulating miRNA levels are solely of renal origin remains unknown. Elevated levels of certain miRNAs in the plasma may be the result of renal cell death associated with leakage of cytoplasmic components or active secretion from the surviving cells, membrane microparticles, exosomes or in protein-bound forms [23]. In a study of atherosclerosis, the delivery of miR-126 by endothelial cell-derived apoptotic bodies was found to be able to induce CXCL-dependent vascular protection [24], indicating that upon injury, apoptosis was switched on and miRNAs could be secreted into extracellular space or circulation in apoptotic bodies. Whether it was the same case in our study needed further study. The whole body is also thought to respond to a renal IRI, and circulating miRNAs might have originated from elsewhere besides the kidney. Circulating miRNAs could play an important role in interorgan communication rather than being mere bystanders of tissue injury [25]. Recent study found microvesicles carrying specific microRNAs released from endothelial progenitor cells exert a protective effect in experimental acute renal IRI [26]. Additional studies are needed to identify specific organ and cell types producing miRNAs.

There were several noteworthy points in our clinical study. Firstly, the plasma level of miR-192 in non-AKI patients at the time of admission to ICU (0 h) was also significantly higher than the preoperation level. This may be associated with cardiopulmonary bypass and cardiac IRI. Although miR-192 level in the heart tissue is much lower than that in the kidney, investigators showed that hypoxia induced miR-192 expression and excretion in the sera of patients with post-acute myocardial infarction (AMI) [27]. Moreover, miR-192 expression pattern in humans and rats seems to be different. The level of plasma miR-192 in AKI patients was significantly higher than that of non-AKI patients at 2 h after ICU admission. However, rat plasma miR-192 began to increase at 3 h after reperfusion. The fact that patients stayed in the anesthesia resuscitate room between cardiac blood flow restoration and ICU admission for a 1- to 2-h period may explain this discrepancy. In addition, only one time point difference in the level of miR-192 was observed and the AUC was not very high. Small number of cases, mild clinical AKI and limited time points between 2 and 24 h after reperfusion may have reduced the diagnostic value of miR-192 in AKI. Future studies with large sample size and intensive time points are warranted. The last thing to be noted was that although the incidence of AKI in our clinical study was similar to other previous studies [28], the severity of AKI developed in this study was relatively mild. Only 4 out of 35 AKI patients were with AKI stage 3, and only one of them needed RRT. That might be due to some risk factors for AKI, such as diabetes, lupus nephritis, infection diseases and pre-existing cancer that were excluded in this study in case of all these confounding factors affected the analysis of miR-192 levels.

In this study, the molecular mechanisms underlying the deregulation of circulating and renal miRNAs could not be determined. Previous studies suggest that miR-192 was linked to both G1 and G2/M cell cycle arrest in other contexts [29, 30] and was frequently downregulated in colorectal cancer, renal childhood neoplasms and multiple myeloma [31–33]. Roy et al. [34] recently found that tumor necrosis factor alpha (TNF-α) was an upstream regulator of miR-192 in acute liver injury and confirmed a protective effect of downregulation of miR-192 in hepatocytes through Zeb2. The exact pathophysiological effect of these changes needs to be further investigated.

The miRNA PCR array also showed that several miRNAs decreased after IRI, which have not yet investigated. The purpose of this study was to identify a miRNA with diagnostic implications for AKI after IRI. Owing to the low abundance of miRNAs in the blood, if miRNAs are further decreased under disease conditions, the level of these miRNAs may be beyond the range of detection of the existing techniques. The mechanism underlying the downregulation of these miRNAs is still not clear and should be investigated. In addition, we observed dynamic changes of plasma miR-192 levels in patients undergoing cardiac surgery and provided a time window for clinical testing.

In summary, this study showed dynamic changes of plasma and kidney miR-192 levels in AKI and plasma miR-192 might be a predictor for ischemic AKI. Larger cohort studies are needed to confirm these findings, and further studies are required to identify the underlying molecular pathophysiological mechanisms.
